# Effect of the Alkyl Density of Acrylic Acid Ester on the Viscosity-Reducing Effect of Polycarboxylate Superplasticizer

**DOI:** 10.3390/molecules28217293

**Published:** 2023-10-27

**Authors:** Yingying Chen, Yujie Chen, Yuan Liu, Jia Tao, Runxia Liu, Ziwei Li, Fei Liu, Min Li

**Affiliations:** 1 Guizhou Provincial Key Laboratory of Green Chemical and Clean Energy Technology, School of Chemistry and Chemical Engineering, Guizhou University, Guiyang 550025, China; 2 China Railway Fifth Bureau Group Co., Ltd., Guiyang 550003, China; 3 Guizhou Tianwei Building Materials Technology Co., Ltd., Guiyang 550025, China; 4 School of Civil Engineering, Guizhou Institute of Technology, Guiyang 550003, China

**Keywords:** polycarboxylate superplasticizer, acrylic acid ester, viscosity-reducing effect, methyl acrylate, cement pastes, concrete

## Abstract

Concrete is vital for the development of modern buildings. However, they suffer from the high viscosity problem in their application process due to the use of a low water–cement ratio in order to maintain their high strength. Developing PCEs with the presence of ester functional groups in their molecular structure is one of the most effective measures to improve the flowability of concrete. Here, three PCEs with different alkyl densities of acrylic acid ester: PCE-M, PCE-E, and PCE-B were designed to explore their viscosity-reducing effect on the performance of cement and concrete. The structures of the three PCEs were characterized via Fourier transform infrared (FTIR) spectra, proton nuclear magnetic resonance (^1^H NMR), and gel permeation chromatography (GPC). Their properties were also determined via zeta potential, surface tension, and rheological experiments. It was found that PCE-M had the best performance, with the lowest surface tension, highest zeta potential, and therefore highest charge density on the cement particles, lowest viscosity, and highest flowability of cement paste, and exhibited the best performance of concrete in terms of workability. The best performance of PCE-M in reducing the viscosity of cement and concrete can be ascribed to the smallest amount of water-repellent alkyl groups, enhancing the electrostatic repulsion and reducing the viscosity, thereby boosting the dispersion and stabilization of cement pastes and concrete. This study shed lights on designing other PCEs with high viscosity-reducing effects via an ester group control.

## 1. Introduction

Nowadays, concrete has become increasingly demanding, especially for the ultra-high performance concrete, which has high strength and good durability due to the rapid development of modern buildings [1,2,3]. However, the high viscosity problem of these concretes, due to their low water to cement ratios and high cement contents, decreases their flowability and workability, hindering their application. Polycarboxylate superplasticizer (PCE), which can reduce the water consumption but maintain the strength of concrete and increase their flowability under a low dosage, has attracted intensive amounts of attention [4,5]. A considerable amount of research has been conducted to develop high-performance PCEs with different backbones, different side chain lengths, and different functional groups in order to improve the fluidity and high strength of concrete at low water to cement ratios during its application process [6,7,8,9,10,11,12,13]. The ester functional group in PCEs has been reported to have various functions when they are applied in concrete, such as slump retention, retardation, and viscosity reduction [14,15,16,17]. For example, the slump-protecting PCE was synthesized via the copolymerization reaction of poly(ethylene glycol) methyl ether methacrylate, acrylic acid (AA), and ester or amide groups by Lin’s group, which not only improves the dispersion performance but also has an excellent slump-protecting effect [6]. It was found that PCEs prepared using esters have better performance than those synthesized using amides. Therefore, they also used trimethylolpropane triacrylate and trimethylolpropane ethoxylate triacrylate to prepare four PCEs containing ester groups [15]. They concluded that PCEs with higher ethoxy group contents exhibited better workability. The better workability of these ester-based PCEs can be attributed to the release of considerable anchoring groups, improving the adsorption of PCE on the surface of cement and concrete and promoting their dispersibility. In another study, an ester-based slow-release PCE, which exhibits good fluidity and excellent slump retention, was also been designed by Feng et al. using isopentenyl polyethylene glycol [16]. It was found that an adequate amount of unsaturated triethyl citrate can be hydrolyzed significantly, leading to their excellent slow-release and dispersive properties. Three different slump-preservation PCEs were designed and synthesized by Pang et al. using methylallyl polyoxyethylene ether (HPEG), isopentenyl polyoxyethylene ether (IPEG), vinyl polyoxyethylene ether, and hydroxyethyl acrylate as the main raw materials [18]. The excellent slump retention performance was ascribed to the steric hindrance of the ester groups. Huang et al. used IPEG, AA, and dimethylaminoethyl methacrylate to prepare PCE with a viscosity-reducing property [13]. Furthermore, hydrophobic alkyl acrylates have a lower cost, which are suitable for industrial applications.

Based on the discussions above, in this paper, the effect of hydrophobic alkyl acrylate esters on the dispersion properties of PCE was explored. Three different PCEs were prepared from HPEG, AA, and monomers containing different ester groups in aqueous solution (methyl acrylate (MA), ethyl acrylate (EA), and butyl acrylate (BA), denoted as PCE-M, PCE-E, and PCE-B, respectively) via free radical polymerization. Their structural characterizations were performed using Fourier transform infrared (FTIR) spectra, nuclear magnetic resonance (NMR), and gel permeation chromatography (GPC) measurements. Zeta potential and surface tension were also measured to investigate the dispersibility of the cement. The rheological properties of cement paste were tested using a rheometer. The dispersion properties on cement and concrete were discussed. Finally, PCE-M, PCE-E, and PCE-B were tested through the applications of cement and concrete.

## 2. Results and Discussion

### 2.1. FTIR Analysis

**Figure 1:**
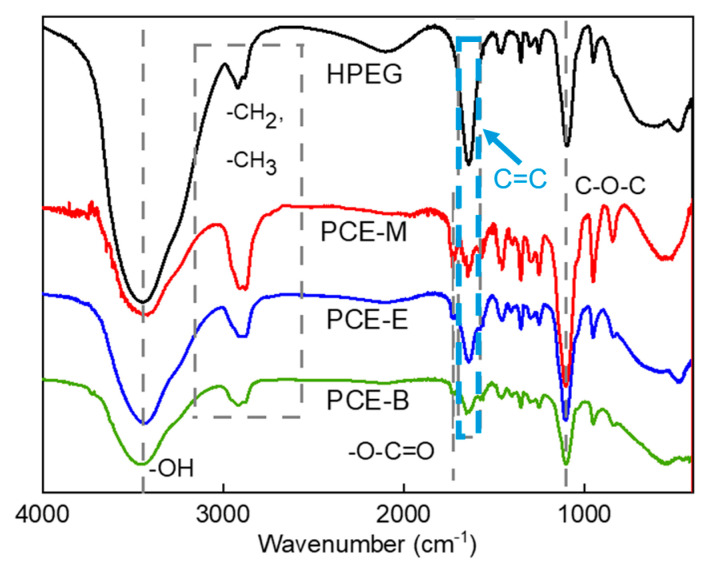
FTIR spectra of the synthesized PCEs.

FTIR analysis was employed to characterize the functional groups in both HPEG and different PCEs so as to confirm the occurrence of the polymerization process. Figure 1 shows the IR spectra of the raw material HPEG and different PCEs: PCE-M, PCE-E, and PCE-B. From Figure 1, it can be seen that the synthesized PCEs contain different functional groups, such as hydroxyl (-OH) and ether (C-O-C) groups, which are similar with those in the polyether monomer HPEG, as well as the ester (-O-C=O) group. Meanwhile, in contrast to HPEG, the intensity of the carbon–carbon double bond (C=C at 1639 cm^−1^) peaks in the PCEs were significantly less [19,20]. In addition, none of the unsaturated C-H absorption peaks appeared in the plots. All of these results indicate that the monomers have largely copolymerized successfully. These functional groups all have characteristic absorption peaks in the infrared spectrum, the positions of which have been essentially fixed in a certain range and shifted differently with the nearby molecular structure. Specifically, 3438 cm^−1^ is the characteristic absorption peak of O-H, while 2914 cm^−1^ and 2876 cm^−1^ are the absorption peaks of the stretching vibration of C-H, 2914 cm^−1^ is the absorption peak in -CH_3_, 2876 cm^−1^ is the absorption peak of -C-H in -CH_2_, and 1728 cm^−1^ and 1100 cm^−1^ can be assigned to the characteristic stretching vibration of C=O and the absorption peak of the C-O-C ether bond in the molecular structure, respectively [21]. These results are in agreement with the study by Ma et al. [22].

### 2.2. ^1^H NMR Analysis

**Figure 2:**
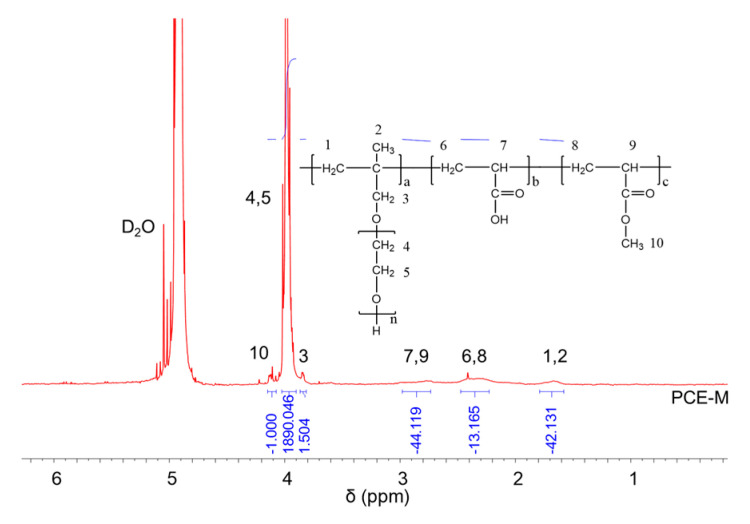
^1^H NMR spectra of PCE-M.

**Figure 3:**
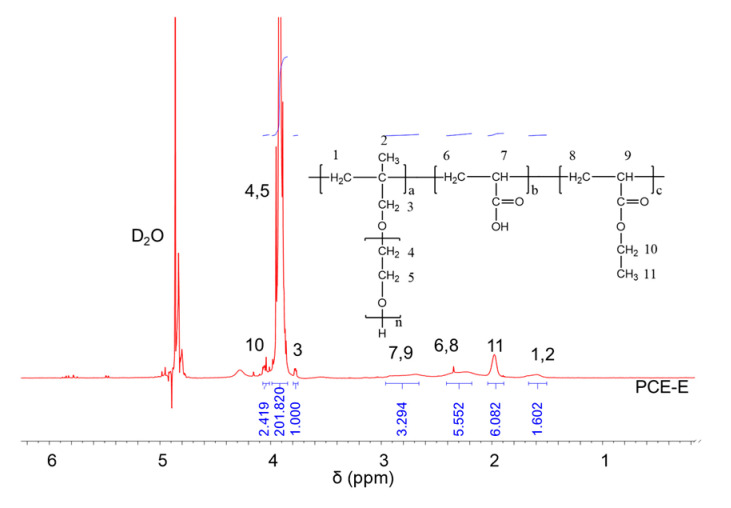
^1^H NMR spectra of PCE-E.

**Figure 4:**
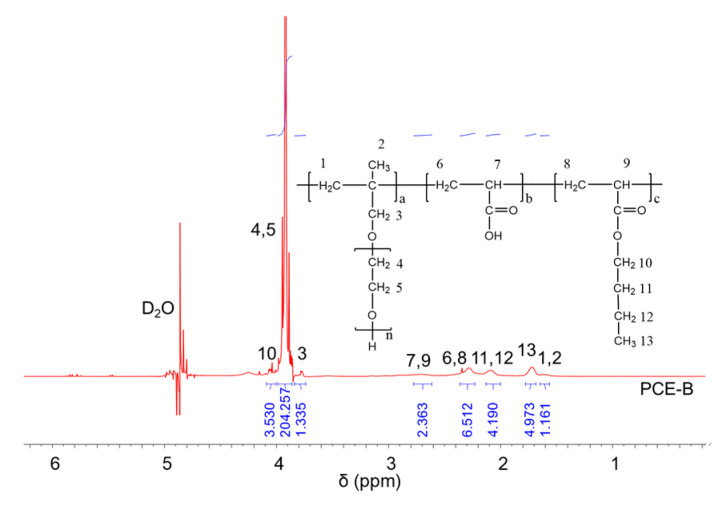
^1^H NMR spectra of PCE-B.

^1^H NMR spectra were carried out to analyze the molecular structures of the synthesized PCEs, as shown in Figure 2, Figure 3 and Figure 4, respectively. It can be seen from Figure 2, Figure 3 and Figure 4 that the ^1^H peak, which has a chemical shift value (δ) of 4.70 ppm, can be attributed to the D_2_O solvent [23]. Additionally, there was no proton peak at δ = 5.30 ppm, which refers to the carbon–carbon double bond for all the three synthesized PCEs, demonstrating that the carbon–carbon double bond has been completely polymerized in the reaction, which corelates well with the IR spectrum, as discussed above [24].

Likewise, the structure of the synthesized PCEs can also be confirmed via different δ values of the ^1^H peaks, suggesting different H atoms at different positions of the PCEs structures. For example, from Figure 2, for PCE-M, H atoms at the positions of 1 (δ = 1.48 ppm), 2 (δ = 0.85 ppm), 3 (δ = 3.65 ppm), 4 and 5 (δ = 3.80 ppm), 6 and 8 (δ = 2.21 ppm), 7 and 9 (δ = 2.73 ppm), and 10 (δ = 3.91 ppm) can be determined. In comparison, as for PCE-E and PCE-B, from Figure 3 and Figure 4, respectively, although the ^1^H peak intensities of H atoms at the positions from 1 to 10 decreased especially for PCE-E, the δ values for these ten peaks were similar, suggesting that all three PCEs have a similar structure at these positions. This is in good accordance with the experimental design of the structures of the three PCEs. However, the appearance of a new peak at δ = 1.86 ppm (position 11) of PCE-E, and new peaks at δ = 1.97 ppm (positions 11 and 12) and δ = 1.60 ppm (position 13) of PCE-B illustrate the successful synthesis of these PCEs with different hydrophobic alkyl acrylate ester groups.

### 2.3. GPC Analysis

**Figure 5:**
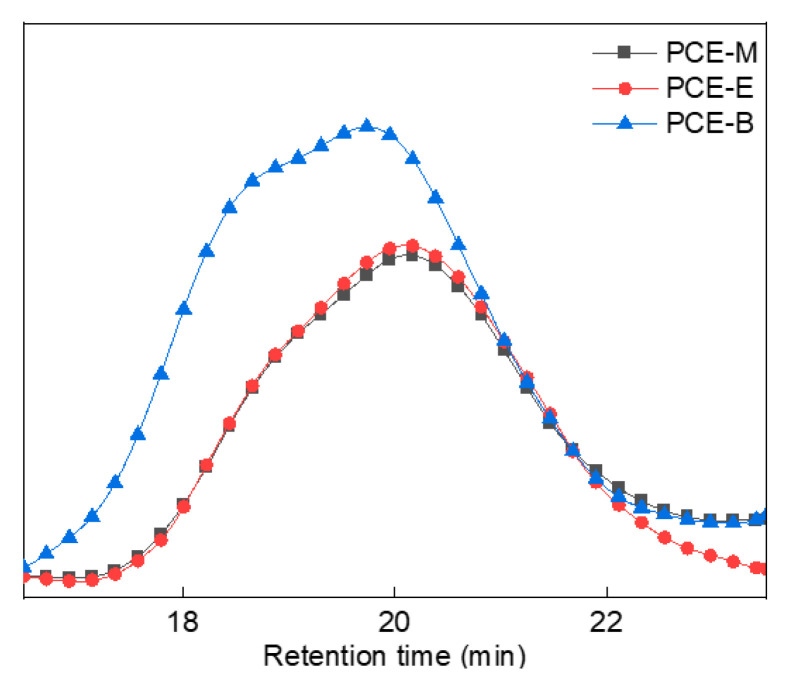
GPC spectra of the synthesized PCEs.

In order to better analyze the molecular weight distribution of the synthesized PCEs, GPC tests were performed, as shown in Figure 5. GPC data, which include the values of the number average molecular weight (M_n_), the heavy average molecular weight (M_w_), the z-average molecular weight (M_z_), and the dispersibility index of molecular weight (PDI, M_w_/M_n_), were described in Table 1. From Figure 5 and Table 1, it can be observed that all the synthesized PCEs have comparable PDI values (2.0–2.5) with previous studies [20,25,26], suggesting that they have relatively narrow molecular weight distributions and good homogeneous stability. In addition, the M_w_ of the PCEs increased with the increase in the alkyl density of acrylic acid ester.

### 2.4. Surface Tension

**Figure 6:**
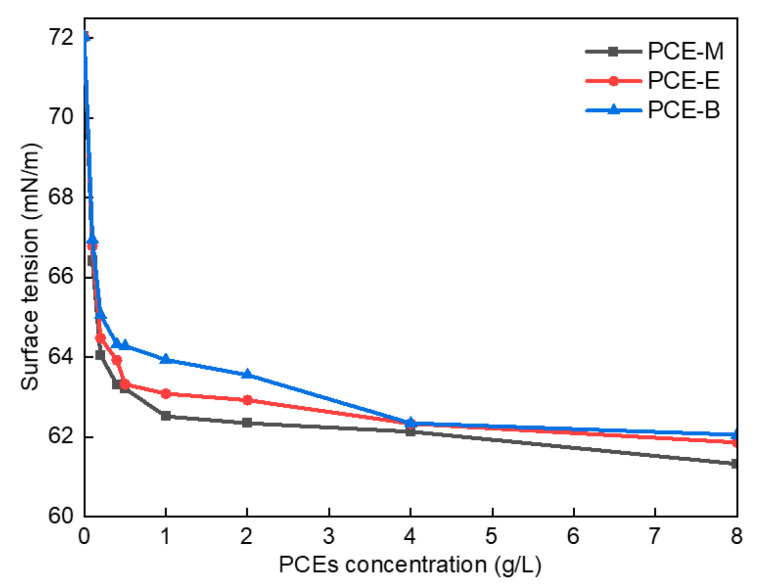
Surface tension of the synthesized PCEs.

Figure 6 shows the surface tension diagram of different PCEs. As shown in Figure 6, the surface tension of the deionized (DI) water was 72.03 mN/m when no PCE was added. In comparison, following the addition of a small amount of PCE-M, PCE-E, and PCE-B, their surface tension decreased significantly to 66.41 mN/m, 66.79 mN/m, and 66.95 mN/m, respectively. The lowest surface tension of PCE-M can be attributed to the fact that MA has the smallest polarity and molecular weight, which weakens intermolecular interaction forces and leads to its lowest surface tension. Further, with the increase of the concentrations of the PCEs, their surface tensions correspondingly decreased. This can be due to the introduction of the hydrophobic ester groups into the PCEs, bringing about an imbalance in the forces on the surface of the water molecules, thereby resulting in a decrease in the surface tension of the PCE solution [27]. At low concentrations, the surface tension of the PCEs decreased more significantly. At the PCE solution concentration of around 4 g/L, the surface tension decreased to its maximum extent. Finally, the equilibrium values of 61.32 mN/m, 61.86 mN/m, and 62.05 mN/m for PCE-M, PCE-E, and PCE-B, respectively, were achieved, with a further increase of the concentrations of the PCEs. This can be as the result of the saturation of the interaction force between molecules at high PCE concentrations, resulting in their difficult access to each other, as well as the equilibrium values of their surface tension [28]. These results suggest that the addition of PCEs is promising to increase the dispersion of cement particles.

### 2.5. Zeta Potential

**Figure 7:**
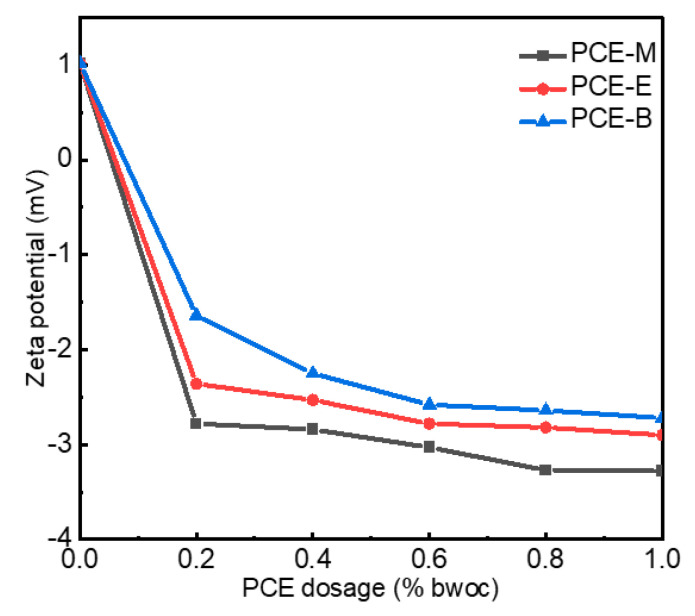
The zeta potential of cement paste with PCEs.

The zeta potential refers to the potential of the shear plane, which is an important index to characterize the stability of a colloidal dispersion system. Non-charged side chains of the PCEs and ions, including Ca^2+^ and SO_4_^2−^, usually form the shear plane of the cement [22,29]. PCEs, which have negatively charged backbones, can be adsorbed on the positively charged cement surface. In order to explore the surface charge density of cement with different PCEs, the zeta potential was performed, as shown in Figure 7. As shown in Figure 7, the zeta potential without the addition of PCEs exhibited positive values of around 1 mV. When only 0.2% PCEs were added, the zeta potential rapidly decreased to −2.75 mV, −2.34 mV, and −1.67 mV for PCE-M, PCE-E, and PCE-B, respectively. With the further increase in PCE dosage to 1%, the zeta potential gradually decreased and reached stable values of −3.28 mV, −2.90 mV, and −2.72 mV for PCE-M, PCE-E, and PCE-B, respectively [30]. Normally, the higher absolute value of the zeta potential, the higher surface charge on the cement surface, and the higher stability of the cement with PCE solutions. The highest absolute value of the zeta potential of PCE-M indicates that it provides the highest amount of anionic charge. This can be attributed to the shortest chain length of MA, making the easier dispersion and reaction with the cement paste [31]. Additionally, competitive adsorption between the adsorbed PCE anion groups and the anions in the cement lead to electrostatic repulsion, breaking the flocculation structure of the cement paste, releasing more free water, and increasing the dispersion of cement [30]. In comparison, the alkyl ethyl acrylate and butyl acrylate in PCE-E and PCE-B reduce the charge density and, thereby, electrostatic repulsion, leading to the decrease of the amount of free water and poor dispersion of the cement.

### 2.6. Rheological Properties

**Figure 8:**
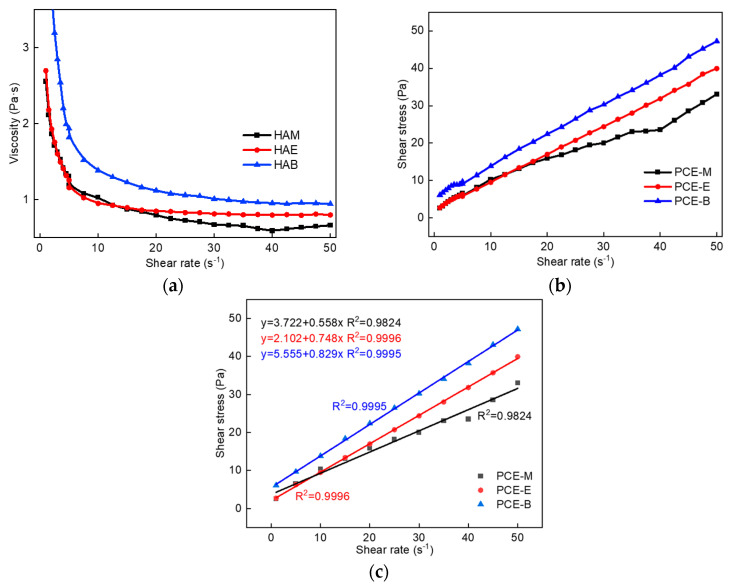
(**a**) The viscosity of cement paste with different PCEs. (**b**) The shear stress of cement paste with different PCEs. (**c**) The fitting results of the rheological parameters of cement paste with different PCEs.

To explore the effect of PCEs with different ester groups on the rheological properties of cement pastes, the plastic viscosity and yield stress changes of three different PCEs were tested, as shown in Figure 8. It can be found from Figure 8a that the viscosity of cement paste with the addition of PCE-M is the smallest under the same shear rate, indicating the highest viscosity-reducing effect of PCE-M, thereby improving the fluidity and pumping effect of concrete. This corelates well with the highest flowability results of PCE-M, which shall be discussed in Section 2.7. Additionally, the shear stress gradually increases with the increase in the alkyl density of acrylic acid ester (Figure 8b), demonstrating that it is the easiest for PCE-M to break the flocculation structure between the cement particles and release free water, so as to improve the cement fluidity. The Bingham model was employed to fit the rheological data of cement pastes with different PCEs, as shown in Figure 8c. A linear relationship was obtained between the shear stress and the shear rate for all the PCEs, which is in consistent with the Bingham model [22,32]. Further, the slope of the fitting curve gradually increases with the increase in the alkyl density of acrylic acid ester, indicating that the plastic viscosity of the cement paste gradually increases, which is consistent with the data shown in Figure 8a.

### 2.7. Performances of Different PCEs in Cement

**Figure 9:**
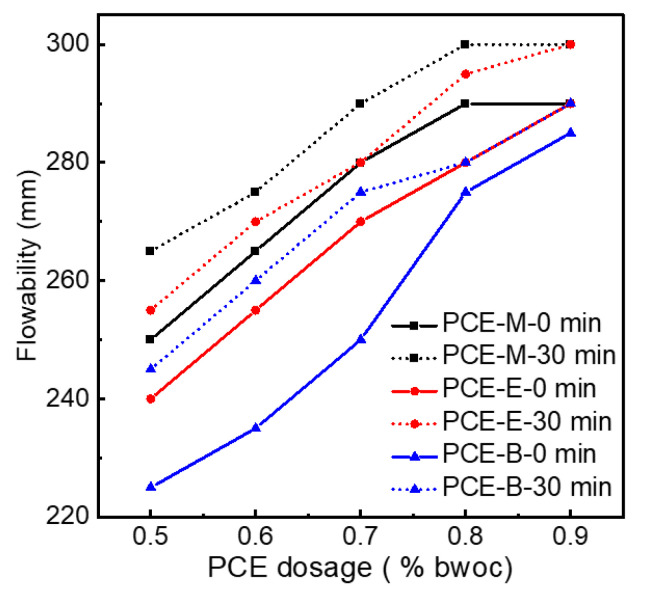
The flowability of the synthesized PCEs.

Figure 9 indicates the initial net cement paste flow and the flowability after 30 min for different PCEs. In order to figure out the effect of PCEs synthesized from different acrylates on the flowability of the cement, PCEs with different dosages were tested. It can be seen from Figure 9 that, for the same type of PCE, the flowability of cement paste increases with the increase in the dosage of PCE. When the dosage reached 0.9%, the initial flowability of 290 mm, 290 mm, and 285 mm for PCE-M, PCE-E, and PCE-B, respectively, were achieved. A further increase in the dosage of the PCEs will lead to the excessive amount of admixture, resulting in no application value other than water secretion. Therefore, experiments with dosages greater than 0.9% are not necessary for all the synthesized PCEs. In addition, it was interesting to find that the flowability after 30 min for all the three PCEs at all dosages even increased and reached 300 mm for PCE-M and PCE-E at the dosage of 0.9%. This can be attributed to the hydrolysis of the ester group at a later stage, allowing the cement to release more free water, thereby increasing the flowability of cement paste [3]. When comparing the flowability of PCEs with different ester groups, it was found that PCE-M exhibited the highest flowability in terms of under initial conditions and after 30 min under the same dosage of PCEs. This can be due to the fact that the increase in the amount of water-repellent methyl groups leads to the weakening of the electrostatic repulsion between the cement particles [33], which is not conducive to the release of free water and results in the decrease in flowability.

### 2.8. Performances of the PCEs in Concrete

In practice, the air content (A_c_), the time to reach 500 mm (T_500_), the slump flow, and the time to empty a slump cone of concrete (T_sf_) can be used as indicators when testing the cohesiveness of concrete. All things being equal, a concrete mix with a short time has a relatively low cohesion when the air content is within a certain range, which facilitates a better application of the concrete mix in the project. Table 2 shows some concrete application data involving the PCEs. It can be seen from Table 2 that, to achieve the same range in slump (230 mm) and slump flow (around 600 mm), the amount of PCEs added tends to increase with the increase in the alkyl density of acrylic acid ester. This trend was also true for other indicators, such as A_c_, T_500_, and T_sf,m_. These results demonstrate that, compared with PCE-E and PCE-B, PCE-M has a better concrete application effect with a soft state and good compatibility. This is consistent with the GPC structural characterization results described above that PCE-M has the lowest molecular weight, which endows it a higher degree of freedom in water so that the molecular chains of PCE-M are more easily able to spread and quickly adsorbed onto the cement particles. Additionally, the lowest surface tension and highest absolute value of the zeta potential of PCE-M made it a high density of surface charge on the cement particles. Further, the lowest viscosity of PCE-M reflected from the rheological tests also contributed to its best concrete performance. The good concrete performance of PCE-M is also in accordance with the flowability results of the cement, as discussed above.

Based on the characterizations and discussions above, it can be concluded that, compared with PCE-B and PCE-E, PCE-M exhibited the most profound viscosity-reducing effect. This can be attributed to its shortest side chain length and its lowest molecular weight, as confirmed from GPC analysis, yielding its highest molecular freedom to be adsorbed on the cement surface via electrostatic interactions and weakening the intermolecular interaction forces, thereby resulting in its lowest surface tension. Additionally, PCE-M showed the highest amount of anionic charge on the surface of cement particles, as revealed by its highest absolute value of the zeta potential, generating a high adsorption amount and promoting electrostatic repulsion between the cement particles. Further, the low plastic viscosity and yield stress of PCE-M rendered a small steric hindrance of it to break up its flocculent structure and release more free water due to the ester groups. All of these factors reduced the viscosity and increased the dispersion and flowability of cement particles and the workability of concrete.

## 3. Materials and Methods

### 3.1. Materials

Isobutene polyethylene glycol ether (HPEG), with a molecular weight of 2400, was supplied by Dongke Company, China. Ammonium persulfate (APS, 98.5%), 3-mercaptopropionic acid (MPA, 99%), acrylic acid (AA, 99%), methyl acrylate (MA, 98.5%), ethyl acrylate (EA, 98%), butyl acrylate (BA, 99%), sodium hydroxide (NaOH, 96%), and deuterium oxide (D_2_O, 99%) were purchased from Macklin Chemical, China. The reference cement was supplied by Fushun Aosaier Co. (Liaoning, China), and its specifications are presented in Table 3.

### 3.2. Synthesis

**Figure 10:**
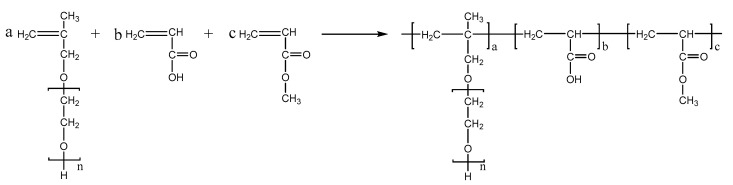
The synthesis of PCE-M.

**Figure 11:**
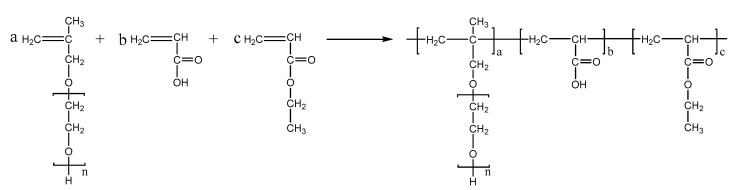
The synthesis of PCE-E.

**Figure 12:**
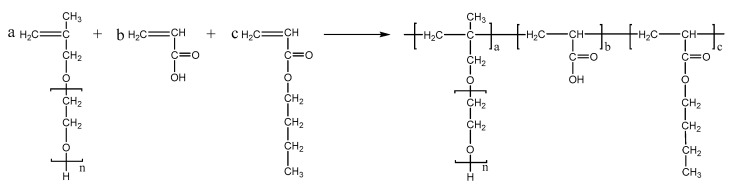
The synthesis of PCE-B.

PCEs, with different acrylic ester groups, were synthesized via the solution polymerization method. Specifically, the macromonomer HPEG and some water were poured into a three-necked flask equipped with a stirring paddle and a thermometer. Subsequently, the three-necked flask was put into a stirrer and stirred at a constant temperature of 30 °C for 20 min. After the temperature was increased to 80 °C, solution A (0.0982 mL MPA and 0.1815 g APS) and solution B (4.1 mL AA and 3.4 mL MA) were added into the flask using a syringe pump and the solution was kept stirring at 80 °C for 1.5 h. After the reaction was completed, the pH of the polymer solution was adjusted to 6–8 using a NaOH solution (30%). When the reaction temperature was cooled to room temperature, the highly dispersed PCEs, with different acrylic ester groups synthesized from MA, EA, and BA, were obtained and denoted as PCE-M, PCE-E, and PCE-B, respectively. The reaction equations are shown in Figure 10, Figure 11 and Figure 12 below.

### 3.3. Test Methods

#### 3.3.1. FTIR Measurements

The FTIR spectra (Nicolet iS50, Thermo Fisher Scientific, Waltham, MA, USA) was used to characterize the functional groups on different PCEs. Firstly, the synthesized products were freeze dried at −80 °C for 24 h using a freeze dryer (LGJ~10A~80T, Shanghai Shunzhi Fertilizer Machinery, Shanghai, China.). Secondly, PCEs solutions with 10% solid content were prepared and determined using a refractometer (LB32T). Subsequently, 0.1 g of KBr was pressed under 15 MPa to make it as a tablet. Finally, 10 μL of different PCE solutions were dropped on the surface of KBr for analysis at the absorption wavelength range between 400 cm^−1^ and 4000 cm^−1^.

#### 3.3.2. NMR Measurements

The structure of the synthesized PCEs was confirmed using a 400M NMR spectrometer (JNM-ECZ400S/L1, JEOL, Tokyo, Japan.). After drying at a low temperature for 24 h, they were dissolved in a D_2_O deuterated reagent, which was used as the solvent. After transferring to a nuclear magnetic tube, the ^1^H NMR spectra were analyzed at room temperature to determine the chemical shift values of different hydrogen atoms in the PCEs.

#### 3.3.3. GPC Measurements

LabSolutions GPC was used to determine the number average molecular weight (M_n_), weight average molecular weight (M_w_), and polydispersity index (PDI) of the synthesized PCEs. The synthesized product was dried under a low temperature for 24 h and prepared into a sample with a concentration of 1.0 mg/mL. Then, 40 μL of the solution was injected and a 0.4% NaNO_3_ aqueous solution was used as the mobile phase, with a flow rate of 1.0 mL/min.

#### 3.3.4. Surface Tension Measurements

The surface tension of the polycarboxylate superplasticizer solution was measured on an OSA60 microsurface contact angle tester, with a measurement range and accuracy of 0.01–2000 mN/m and ±0.01 mN/m, respectively. The products were dried under a low temperature and made into different concentration with 0 g/L, 0.2 g/L, 0.4 g/L, 0.5 g/L, 1.0 g/L, 2.0 g/L, 4.0 g/L, and 8.0 g/L. Generally, the measurements of the surface tension of the PCE were repeated three times at 25 °C, and the average value of the three measurements was calculated as the final value.

#### 3.3.5. Zeta Potential Measurements

The zeta potential of polycarboxylate superplasticizer cement pastes with different dosages of 0%, 0.2%, 0.4%, 0.6%, 0.8%, and 1.0% at 25 °C were characterized on the zeta potential and nano-particle size analyzer (Delsa ^TM^ Nano C, Beckman Coulter, Brea, CA, USA). At a water–cement ratio of 800:1, 1 g of reference cement was poured into different beakers containing 800 mL of DI water and different dosages of polycarboxylate superplasticizers to prepare the diluted cement paste. After stirring for 5–10 min, followed by the subsequent standing period, the supernatant of the cement paste was taken to measure the zeta potential on the surface of the cement particles with different dosages of polycarboxylate superplasticizer. Three measurements were repeated at each dosage, and the average value was taken as the final zeta potential.

#### 3.3.6. Performances of the Polymers in Cement

The fluidity measurements of cement pastes containing different PCEs were performed according to the National Standards of China (GB/T 8077-2012) [34]. The amount of water required for a water–cement ratio (W/C) of 0.29 was determined by subtracting the water contained in the PCE solution from 87 mL of DI water. Specifically, 300 g of cement was mixed with the calculated amount of DI water and stirred at a low and high speed for 2 min. Subsequently, the mixture was transferred into a truncated cone with the dimensions of 60 × 36 × 60 mm on a glass plate. Then, 30 s after vertically removing the cone, the diameter of the spread paste was measured at different positions. Similarly, the fluidity of cement after 30 min of placement was also tested. All measurements were repeated twice, and the average value was selected as the paste fluidity.

#### 3.3.7. Rheological Property Measurements

Rheological properties, such as yield stress and plastic viscosity, were determined using a rheometer (ARES-G2, TA Instruments, Newcastle, Delaware, USA). The cement pastes were prepared using the method described in Section 3.3.6. After pouring the pastes into the rheometer, in order to mitigate the influence of applied stress, the samples were pre-sheared at a shear rate of 50 s^−1^ for 1 min. Subsequently, the shear rate was decreased step by step to 0 s^−1^, with a pause of 1 min in each speed. At the last 5 s, the shear stress and shear rate were taken. The rheological properties was described using Equation (1):

(1) $$\tau =\tau_{0}+K{\dot{\gamma }}^{n}$$

where τ, τ_0_, $\dot{\gamma }$, K, and *n* refer to the shear stress (Pa), yield stress (Pa), shear rate (s^−1^), consistency coefficient (Pa·s*^n^*), and rheological index, respectively.

The equivalent plastic viscosity was calculated using Equation (2):

(2) $$\mu =\frac{3K}{n+2}{\dot{\gamma }}_{max}^{n-1}$$

where *μ* and ${\dot{\gamma }}_{max}$ indicate the equivalent plastic viscosity (Pa·s) and the maximum shear rate, respectively.

#### 3.3.8. Performances of the Polymers in Concrete

The performances of the PCEs in concrete, such as the slump flow, expansion flow, air content (Ac), time to reach 500 mm in diameter (T_500_), and time to empty the inverted slump cone, were also tested by referring to the relevant test methods in GB8076-2008 [35] Concrete Admixture and GB/T50080-2016 [36] “Standard for Performance Test Methods of Ordinary Concrete Mixes”.

## 4. Conclusions

Three PCEs with different alkyl densities of acrylic acid ester: PCE-M, PCE-E, and PCE-B have been prepared to explore the effect of different ester-based monomers on the performance and applications of cement and concrete. Structural characterizations, such as FTIR and ^1^H NMR, as well as property measurements for instance surface tension, zeta potential, and rheological experiments, have been performed to better understand the flowability and concrete performance of these PCEs. It was found that the greater the methyl content of the acrylate, the smaller the decrease in surface tension, i.e., PCE-M > PCE-E > PCE-B; the smaller the decrease in the zeta potential, i.e., the absolute magnitude of the zeta potential was PCE-M > PCE-E > PCE-B. In addition, the flow of the cement paste with the three PCEs was always PCE-M > PCE-E > PCE-B, both initially and after 30 min. The T_500_, A_c_, and T_sf_,_m_ measured for the three PCEs after being added to the concrete in application tests also showed the similar trend: PCE-M < PCE-E < PCE-B. These results suggest that, with the smallest amount of water-repellent alkyl groups, PCE-M has the most profound influence on reducing the viscosity and increasing the flowability of cement, as well as enhancing the performance of concrete in terms of workability. The outstanding water-reducing effect of PCE-M can be attributed to the water-repellent groups in increasing the surface charge density and intermolecular electrostatic repulsion evidenced from the zeta potential data, reducing the viscosity and steric hindrance effect of cement paste, proved from the rheological experiments, etc., enhancing the dispersion and stabilization of cement pastes and concrete. This study provides ideas in studying and designing other PCEs with a high viscosity-reducing effect for both cement and concrete via an ester group control.

## Figures and Tables

**Table 1:** GPC data of different PCEs.

PCE	M_n_	M_w_	PDI
PCE-M	25,798	65,500	2.54
PCE-E	28,639	66,100	2.31
PCE-B	26,936	67,246	2.50

**Table 2:** The test data of concrete mixtures of PCEs.

PCE	Dosage (% bwoc ^1^)	Slump/Slump Flow (mm)	A_c_ (%)	T_500_	T_sf,m_^2^
PCE-M	1.70	230/590	3.3	4.4	4.78
PCE-E	1.75	230/600	5.0	5.13	4.82
PCE-B	1.80	230/610	9.0	5.31	5.22

^1^ bwoc means by weight of cement; ^2^ T_sf,m_ stands for the average time for two experiments of emptying a slump cone of concrete.

**Table 3:** Chemical compositions and physical properties of the reference cement.

Oxide	wt%	Physical Properties	
SiO_2_	20.40	Density (g/cm^−3^)	3.11
Al_2_O_3_	4.40	Specific surface area (m^2^/kg)	356
Fe_2_O_3_	3.27	Setting time (min)	
CaO	62.70	Initial	141
MgO	2.86	Final	200
SO_3_	2.18	Compressive strength (MPa)	
NaOeq	0.59	7 days	37.7
f-CaO	0.78	28 days	50.8
Loss	1.75	Flexural strength (MPa)	
Cl^−^	0.018	7 days	7.2
Total	98.948	28 days	8.6

## Data Availability

Not applicable.

## Sample Availability

Samples of the compounds are not available from the authors.
